# Night Terrors and Delirium in a Child With Presumed Rapid-Onset Obesity With Hypothalamic Dysfunction, Hypoventilation, and Autonomic Dysregulation

**DOI:** 10.7759/cureus.107609

**Published:** 2026-04-23

**Authors:** Rachel A Donaldson, Shiva Kothari, Andrea Diaz Stransky, Aishwarya Rajagopalan, Debra E Weese-Mayer

**Affiliations:** 1 Psychiatry, Duke University School of Medicine, Durham, USA; 2 Neurology, Northwestern University Feinberg School of Medicine, Chicago, USA

**Keywords:** anxiety, delirium, night terrors, rohhad, sleep disorders

## Abstract

Comorbid neuropsychiatric conditions, including major depressive disorder, anxiety, bipolar disorder, and psychosis, are reported among children with rapid-onset obesity with hypothalamic dysfunction, hypoventilation, and autonomic dysregulation (ROHHAD). The association of ROHHAD with parasomnias is poorly understood. We describe the presentation and management of night terrors superimposed on delirium in a child with presumed ROHHAD. The child developed an acute altered mental status and increased anxiety during a hospitalization for sepsis and pulmonary emboli, three years after initial rapid-onset obesity. She also had nocturnal-onset limb thrashing and distress beginning an hour after falling asleep and lasting up to several hours, diagnosed as night terrors. Delirium and anxiety improved with quetiapine and clonidine, while the severity of her night terrors improved with anticipatory awakening and haloperidol. Children with ROHHAD may be at risk for night terrors, and their symptoms may present atypically. Early recognition and individualized management may improve safety and quality of life for these children.

## Introduction

Rapid-onset obesity with hypothalamic dysfunction, hypoventilation, and autonomic dysregulation (ROHHAD) is an ultrarare condition that typically presents with rapid-onset weight gain of 20-30 pounds over a three- to 12-month period, followed by phenotypic features typically occurring in the order of the acronym, after a period of normal development in children between ages one and seven years [1]. Although central hypoventilation is a hallmark of ROHHAD, a subset of patients may have preceding obstructive sleep apnea. The hypothalamic dysfunction may include disordered water/salt balance, growth hormone insufficiency, hypothyroidism, and more. The autonomic dysfunction may include altered temperature regulation, vasomotor tone, and more [2].

In the early literature, even before the application of the acronym ROHHAD, published case reports of this phenotype described several neuropsychiatric features, including mood disorders such as anxiety or depression, personality changes, and, if not promptly identified and provided with artificial ventilation, developmental delay, seizures, and sleep disturbances [3-6]. Previously reported sleep-related disturbances in ROHHAD include a child with insomnia and auditory hallucinations and a child with narcolepsy [7,8]. In addition, children with ROHHAD often have comorbid obstructive sleep apnea, altered slow-wave sleep, and mood dysregulation, which are known risk factors for night terrors [9]. To our knowledge, there are no published case reports or case series of night terrors in children with ROHHAD. Delirium also affects many hospitalized children, particularly those in intensive care settings where risk factors such as disrupted sleep, mechanical ventilation, and deliriogenic medications are common [10]. We present a case of behavioral disturbance found to be a combination and superimposition of night terrors and delirium in an early childhood girl with presumed ROHHAD during an admission for pulmonary emboli and sepsis.

## Case presentation

We present the case of a five-year-old girl with a past medical history of rapid-onset weight gain (obesity) beginning in the toddler years after a viral-like illness, with a 50-pound gain documented over six months. At that time, thyroid function tests were normal, and a 1 milligram (mg) dexamethasone suppression test was appropriately suppressed. However, her linear growth velocity increased from the 35th percentile at birth to the 99th percentile at two years, suspected secondarily to weight gain. She was limited to 900 calories daily per the recommendation of Pediatric Endocrinology, but rapid weight gain continued with hyperphagia. She subsequently developed chronic hypernatremia, hypoventilation, and hyperthermia, with a baseline temperature around 38 degrees Celsius. Developmentally, she reached all milestones appropriately. Genetic testing for Prader-Willi syndrome, congenital central hypoventilation syndrome (PHOX2B), and whole exome sequencing were all negative. A cerebrospinal autoimmune encephalopathy panel was negative. Computed tomography of the brain without contrast was normal. Neural crest tumor screening, including periodic urine vanillylmandelic acid, chest X-ray, and abdominal ultrasound, was also negative. A bi-level positive airway pressure (BiPAP) titration sleep study identified an apnea/hypopnea index of 7.9 events per hour diagnostic of sleep apnea, but without hypercarbia, with transcutaneous carbon dioxide values ranging from 33 to 39 millimeters of mercury (mmHg). Three years after initial rapid-onset obesity, her prolactin was elevated at 71.92 nanograms per milliliter (ng/mL), compared to the reference range of 3.34-26.72 ng/mL. She had no family history of rapid-onset or severe obesity. Given her clinical features and after carefully considering alternative diagnoses, she was diagnosed with presumed ROHHAD. This was based on the definition of ROHHAD being rapid-onset weight gain of 20-30 pounds over a three- to 12-month period in previously healthy children, followed by phenotypic features typically occurring in the order of the acronym between one and seven years of age [1-5].

**Figure 1:**
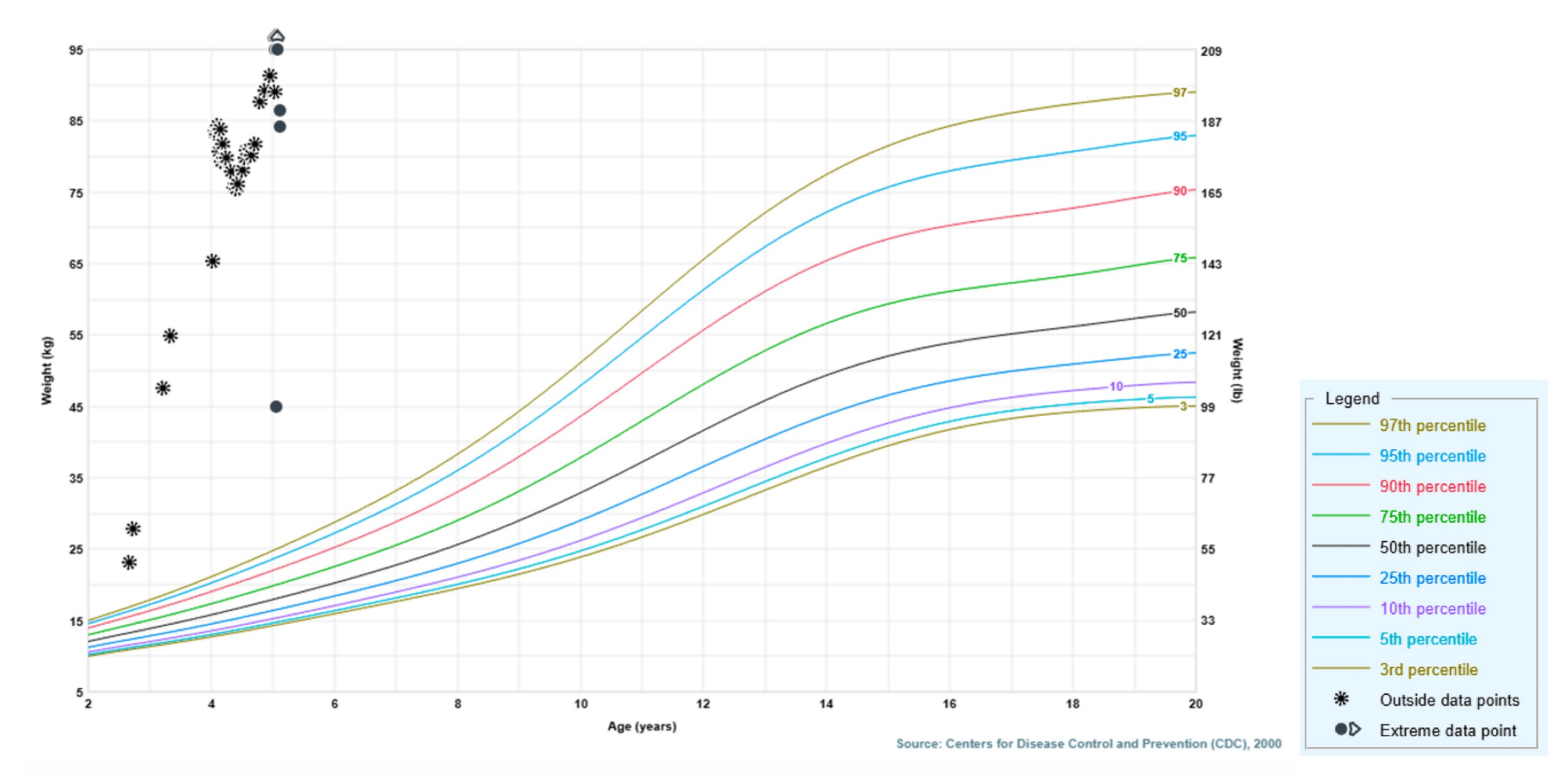
Weight-for-Age Percentile Curve of a Five-Year-Old Child With Presumed ROHHAD kg: kilograms, lb: pounds, ROHHAD: rapid-onset obesity with hypothalamic dysfunction, hypoventilation, and autonomic dysregulation

About three years after initial rapid-onset obesity, she was admitted from home for abrupt-onset tachypneic respiratory distress with a respiratory rate in the low 40s (breaths per minute) and fever to 40.8 degrees Celsius, after a week of abdominal pain, diarrhea, and malaise. Prior to presentation, while at home, she was managed with BiPAP pressures at 20/12 mmHg for overnight sleep. On presentation, she was found to have bilateral pulmonary emboli and sepsis. The respiratory failure with worsening acidosis (i.e., last pH of 6.99 on BiPAP) was managed with intubation and mechanical ventilation. During this hospitalization, despite some weight loss from 89.0 to 86.4 kilograms (kg), she remained >99th percentile weight-for-age throughout the hospitalization. Figure 1 displays her growth curve by weight-for-age percentile, illustrating early, rapid, and severe weight gain consistent with her ROHHAD diagnosis. Her hospital course focused on management of pulmonary emboli via heparin drip, as well as addressing chronic wounds as a possible infectious source under the guidance of Infectious Disease.

**Figure 2:**
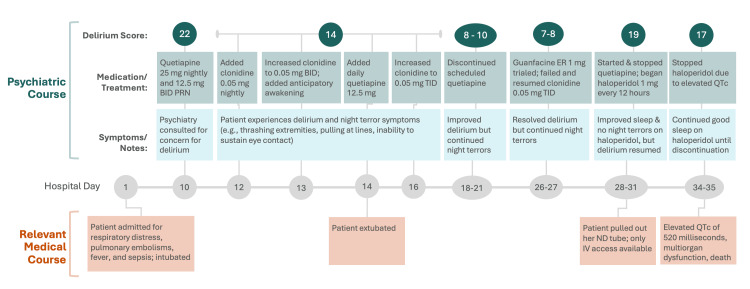
Timeline of Psychiatric Course and Relevant Medical Course in a Five-Year-Old Child With Presumed ROHHAD BID: twice per day, ER: extended-release, IV: intravenous, mg: milligram, ND: nasoduodenal, PRN: as needed, QTc: corrected QT interval, ROHHAD: rapid-onset obesity with hypothalamic dysfunction, hypoventilation, and autonomic dysregulation, TID: three times per day

Figure 2 details a timeline of the patient’s relevant medical course, as well as psychiatric course throughout this hospitalization, including chronicity of medication changes and concurrent observed behavior. On the 10th day of her admission, Psychiatry was consulted due to concern for delirium with an acute change of mental status from her baseline, with new inability to sustain eye contact and inconsistency following commands or answering questions via hand squeeze (patient was intubated). She also appeared anxious, at times tearful, and pulling at intravenous (IV) lines, and, per caregiver report, appearing to stare at things in the room that others could not see. Nursing employed soft restraints on the bilateral hands to prevent her from dislodging her endotracheal tube, nasoduodenal tube, and IV lines. At night, symptoms included insomnia, moving her arms and legs purposelessly, and pulling at lines. Her Cornell Assessment of Pediatric Delirium (CAPD) score, a validated screening tool for pediatric delirium, was 22, well above the threshold score of 9 for screening positive. Her past psychiatric history included generalized anxiety disorder and night terrors. Specifically, night terrors had onset in early childhood and had been ongoing for one and a half years, typically occurring between 12:00 and 4:00 AM and lasting from 15 minutes to three hours. At home, they were characterized by vocalized screaming.

The differential diagnosis for her nocturnal psychiatric presentation includes delirium, night terrors, nightmares, periodic limb movement disorder, medication withdrawal, and hypoxia-associated agitation. Nightmares were considered less likely as they are typically experienced in the last third of the night, compared to the patient’s symptoms, which occurred in the first third of the night. Additionally, children with nightmares are typically able to be woken up and consoled during the episode, and thrashing is uncommon, unlike our patient, whose symptoms were persistent, and thrashing was a key feature. Periodic limb movement disorder can also cause limb movement during sleep; however, it is typically more rhythmic and specific to the lower extremities, which was not the case for our patient. Medication withdrawal is unlikely, as she had not discontinued any medications in the days preceding initial presentation. Hypoxia-associated agitation was not consistent with her presentation, as symptoms were not specifically correlated with periods of hypoxia.

The patient was diagnosed with delirium, a primarily clinical diagnosis, based on several criteria. First, she screened positive for delirium via her CAPD score, which considers eye contact, recognition of caregivers, communication, and awareness. She additionally demonstrated several features consistent with delirium, including acute-onset symptoms, waxing and waning altered consciousness, and possible hallucinations. Additionally, she had multiple risk factors for delirium, including respiratory failure, infection, and administration of deliriogenic medication, including fentanyl. Her anxiety was also believed to be contributory to behaviors, particularly given her history of anxiety prior to admission and caregiver collateral regarding her tendency to be less amenable to care when anxious to avoid triggers from her previous hospitalization.

Her nocturnal constellation of symptoms was distinct from her daytime presentation. Specifically, she showed increased physical manifestations of behavioral distress at night, such as repetitive head movements, grabbing at toys and medical equipment, and thrashing her extremities, with onset around midnight. While these were suggestive of agitated delirium, several factors suggested night terrors, also a primarily clinical diagnosis, distinct from her daytime delirium. These included a clear, acute onset during the first third of the sleep period, episodic nature, persistent and purposeless limb thrashing, inconsolability, and caregiver report of consistent appearance with home episodes that additionally included vocalizations during similar sleeping hours. Thus, she was diagnosed with night terrors, recognizing a differing presentation from home in the setting of intubation and use of restraints. Additionally, frequent interruptions of sleep while hospitalized (e.g., overnight cares and ambient noise) increased the likelihood of night terrors. No electroencephalogram or polysomnography were conducted during sleep disturbance episodes during the child’s hospital stay. Given her intubated status and background delirium, it was not possible to confirm her recollection of night terror events.

Initial treatment included an atypical antipsychotic, quetiapine 25 mg (0.28 mg/kg) nightly and 12.5 mg (0.14 mg/kg) twice per day as needed (BID PRN) for delirium (Figure 2). Clonidine 0.05 mg (0.0006 mg/kg) nightly for anxiety was started soon after and increased to 0.05 mg BID after one day, given a limited initial response. For sleep symptoms, a behavioral intervention was implemented in which the patient’s caregiver woke the patient at 11:00 PM (within one hour of expected onset of night terror symptoms) and allowed her to immediately fall back asleep. Melatonin was briefly considered as an adjunct for night terror management; however, it was held as the family noted a history of subjective poor response and symptom worsening with melatonin when trialed previously.

Delirium improved, and the onset of nocturnal agitation was delayed in response to the intervention, presenting later in the night, but did not resolve. In the following days, the patient was extubated to BiPAP awake and asleep, and her quetiapine dose was increased for worsening delirium and agitation symptoms (Figure 2; hospital day 14). Anticipatory awakening was continued for night terrors. The frequency of clonidine dosing was later increased for increased anxiety and agitation. With subsequent near resolution of delirium, scheduled quetiapine was gradually discontinued (Figure 2; hospital day 26-27). Although night terrors continued and were described as more similar to at home (e.g., increased vocalization), caregivers did feel that the behavioral intervention was subjectively reducing severity in the following days, she was alert during the daytime, and her pediatric delirium screen remained negative. However, night terrors and insomnia persisted with increased nocturnal awake time. Guanfacine extended-release 1 mg (0.01 mg/kg) was trialed in place of clonidine due to its longer-acting nature, but resulted in worsened insomnia, so clonidine was resumed. Soon after, daytime agitation acutely worsened with vocalizing loudly, pulling at lines, and attempting to throw objects. Her CAPD score abruptly increased to 19 (Figure 2; hospital day 28-31). Therefore, scheduled quetiapine was resumed. Shortly after, the patient pulled her nasoduodenal tube, which was unable to be replaced, limiting the route of administration for subsequent medication to IV. She was then started on haloperidol 1 mg (0.01 mg/kg) IV every 12 hours, as haloperidol is the only antipsychotic available in IV formulation. A summary of psychiatric medications prescribed to our patient, including rationale for use, is presented in Table 1.

**Table 1:** Overview of Medications Used for Behavioral and Mood Symptoms in a Five-Year-Old Child With Presumed ROHHAD, Delirium, and Night Terrors ^1^Discontinued due to worsened insomnia ROHHAD: rapid-onset obesity with hypothalamic dysfunction, hypoventilation, and autonomic dysregulation

Medication prescribed to our patient	Why administered
Quetiapine	Treatment of delirium and anxiety
Clonidine	Treatment of anxiety, agitation, and insomnia
Haloperidol	Treatment of anxiety, delirium, and agitation after the patient lost her nasoduodenal tube and had only intravenous access available
Guanfacine^1^	Treatment of anxiety; longer-acting than clonidine

The child responded well to haloperidol with improved sleep, delirium scores, and behavioral disturbances. She was able to visually track objects, attend to conversation, and respond to auditory cues. Additionally, night terrors completely resolved with her caregiver mentioning that she was sleeping peacefully at night. Although she had increased daytime somnolence while on haloperidol, she also had periods of daytime wakefulness during which she was alert, attentive, and engaged. She tolerated haloperidol well with no akathisia, acute dystonia, tardive dyskinesia, or other extrapyramidal symptoms. However, haloperidol was discontinued after four days, given the onset of multisystem organ dysfunction in the setting of an elevated corrected QT interval (QTc) of 520, corrected to 426 milliseconds (ms) by Framingham correction, significantly above her baseline QTc of 357 ms by Framingham. At this time, her potassium was 3.4 millimoles per liter (mmol/L), her magnesium was 2.7 mg per deciliter (mg/dL), and her calcium was 9.7 mg/dL. She was on no other medications carrying a similar risk of QTc prolongation. After haloperidol was discontinued, her QTc decreased to 313 ms by Framingham, but the patient continued to experience progression of acute medical decompensation. This was presumed to be secondary to sepsis, with hemodynamic instability unresponsive to intubation with maximum ventilatory support, vasoactive agents, and fluid resuscitation, leading to her death one day later.

## Discussion

We present the symptoms and management of night terrors superimposed on delirium in a child with ROHHAD. She was hospitalized for sepsis and pulmonary emboli about three years after initial rapid-onset weight gain, and Psychiatry was consulted for altered mental status and nocturnal thrashing. Her delirium, measured by CAPD score, improved while on quetiapine and clonidine, as did her anxiety, per caregiver and nursing report. Her night terror severity was observed to decrease with initiation of anticipatory awakening and while on haloperidol. It is important to note that the causality of these outcomes cannot be attributed to the interventions implemented, as her hospital course was multifactorial with many confounding factors. These include the distress of her critical illness, sedation and intubation, disrupted sleep while hospitalized, and the inherent fluctuating nature of delirium. Although clinical presentation, history, and risk factors were considered in her diagnosis of night terrors, no polysomnography was conducted during this hospitalization.

ROHHAD may be accompanied by neuropsychiatric symptoms, with an estimated 31.4% of patients having behavioral disorders and 15.7% mood disorders such as major depressive disorder or anxiety [6]. Behavioral changes in ROHHAD can include aggression, emotional lability, anxiety, and hallucinations (Table 2) [3,6,7,11,12]. Sleep disorders are also common, including obstructive or central sleep apnea that can lead to hypersomnolence or nocturnal cyanotic episodes [6]. Few cases in the literature discuss parasomnias in ROHHAD. One previously documented case described a child with ROHHAD who experienced daytime somnolence, visual hallucinations, and intermittent loss of facial muscle tone, diagnosed with narcolepsy with cataplexy [8]. Another case described a child with ROHHAD who presented with severe anxiety, insomnia, and auditory hallucinations who responded to lorazepam, olanzapine, and citalopram [7]. However, there is little known regarding the presentation or management of night terrors in ROHHAD. In an article in the Hopkins Children’s magazine, one three-year-old child with ROHHAD was informally described as having experienced night terrors during her initial month of rapid-onset weight gain, although her subsequent clinical course, management, and outcomes were not documented [13]. No peer-reviewed literature to date documents night terrors in ROHHAD. We present a case that demonstrates how night terrors may present differently in ROHHAD (e.g., atypical duration and modified symptoms if restrained or intubated) in these children.

**Table 2:** Cases of ROHHAD in the Literature With Behavioral Descriptions and Psychiatric Medications Prescribed ROHHAD: rapid-onset obesity with hypothalamic dysfunction, hypoventilation, and autonomic dysregulation

Age and gender	Behavioral manifestations	Psychiatric medication used
14-year-old female [7]	Anxiety, insomnia, and auditory hallucinations	Lorazepam, citalopram, and olanzapine
Five-year-old female [11]	Perseverative behavior, reduced flexibility, and anxiety	Cyclophosphamide and amphetamine-dextroamphetamine
Three-year-old male [11]	Perseverative behavior, reduced flexibility, and anxiety	Rituximab and cyclophosphamide
Eight-year-old male [12]	Aggression and impulsivity	Fluoxetine and aripiprazole

Night terrors in typical children present as episodes of screaming, crying, or thrashing, usually lasting seconds to minutes [9]. Risk factors include fever, obstructive sleep apnea, stress, disrupted sleep schedule, and mood dysregulation, many of which are comorbid in children with ROHHAD [9]. These factors suggest that children with ROHHAD may be at higher risk for night terrors. Children with ROHHAD have also been found to have suppressed slow-wave activity associated with their hypothalamic dysfunction [14]. Notably, night terrors occur in a transitional state between sleep and wakefulness during slow-wave sleep; therefore, theoretically, disrupted slow-wave sleep may further increase risk in these children [9].

It is notable that night terror symptoms may present atypically in children with ROHHAD, for example, with reduced vocalization if on BiPAP or intubated, as seen here. Stressors associated with hospitalization in an intensive care unit, including sleep fragmentation, nocturnal cares, loud ambient noise, and medication side effects, may also have exacerbated the night terror symptoms experienced by our patient. Our patient’s night terrors lasted up to several hours, significantly prolonged versus the classic presentation, which could delay diagnosis in some patients with ROHHAD. Features that may differentiate night terrors from delirium include acute onset in the first third of the sleep period, persistent limb thrashing, or a history of similar, frequent episodes. Anticipatory awakening, which is typically first-line, reduced the severity of our patient’s night terrors. Her night terrors persisted even when delirium was well controlled, highlighting the importance of treating each separately. Additionally, night terrors worsened during periods of insomnia (e.g., guanfacine trial) and improved during periods of improved sleep (e.g., haloperidol trial), demonstrating potential benefits of medically optimizing sleep to prevent night terrors in children with ROHHAD.

Limitations include diagnosis and hospitalization at an institution without a dedicated care center for children with ROHHAD, limiting the extent of the phenotypic workup that was done. The patient’s significant medical course during hospitalization with associated medical sequelae could potentially confound some psychiatric symptoms, as there could be an overlap between the two. Bedside nurses and guardian observations helped partly mitigate that limitation. Concurrent treatment of night terrors and delirium may limit clarity of each treatment’s effect. However, the timing of her response to each intervention helped differentiate delirium from night terrors.

## Conclusions

We propose that children with ROHHAD may be at increased risk for developing night terrors versus typically developing peers, based on the patient we present. We present the first formal case report of night terrors in a child with ROHHAD. Further research and case reports are needed to better understand the potential relationship between night terrors and ROHHAD. Night terrors may present non-classically in children with ROHHAD, with prolonged duration, increased thrashing, and/or altered vocalizations, as seen in our patient. Onset in the first three to four hours of the night, consistency between episodes, and the acute nature of episodes may help differentiate night terrors from delirium. A multifactorial approach to treatment is important, considering anticipatory awakening, optimizing sleep quality, and treating underlying delirium where present.
